# 

**Published:** 1892-09

**Authors:** 


*The following donations have been received since the publication of the List in June:*

*August* 31*st*, 1892.

| | |
|---|---|
| Barclay J. Baron, M.B. (1) ... ... ... ... | 7 volumes. |
| G. G. Corbould. (2) ... ... ... ... ... ... | 14 ,, |
| Christopher Elliott, M.D. (3) ... ... ... | 11 ,, |
| H. W. Freeman. (4) ... ... ... ... ... ... | 1 volume. |
| L. M. Griffiths. (5) ... ... ... ... ... ... | 2 volumes. |
| Percy Mathews, M.D. (6) ... ... ... ... | 2 ,, |
| J. Greig Smith. (7) ... ... ... ... ... ... | 41 ,, |

### FIFTH LIST OF BOOKS.

The figures in brackets refer to the figures after the names of the donors, and show by whom the volumes were presented. The books to which no such figures are attached have either been bought from the Library Fund or received through the *Journal.*

---

**Althaus, J.** ... *Medical Electricity* ... ... ... ... (1) 3rd Ed. 1873

**Aretæus** (The Cappadocian.) Περὶ αἰτιῶν καὶ σημείων . . . . acutorum et diuturnorum morborum, &c. (2) 1731

**Bennett, J. H.** .... *Text-Book of Physiology.* ... ... (1) Part II. 1871
**Bergonié et E. J. Moure, J.** *Du Traitement par l'Électrolyse des Déviations et Éperons de la Cloison du Nez* ... ... ... 1892
**Bigg, H.** ... ... *Artificial Limbs* ... ... ... ... ... ... (3) 1885
**Billroth, T.** ... *Surgical Pathology and Therapeutics.* 4th Ed. (Tr. by C. E. Hackley) ... ... ... (1) 1871
**Bourneville, Dr.** . . *Recherches sur l'Epilepsie, l'Hysterie, et l'Idiotie.* Vol. II. 1891
**Bowman, R. B. Todd and W.** *Physiological Anatomy.* (3) 1845; (3) Part III. 1847
**Buxton, D. W.** ... *Anæsthetics* ... ... ... ... ... ... 2nd Ed. 1892

**Cooper and F. S. Edwards, A.** *Diseases of the Rectum and Anus.* 2nd Ed. 1892
**Cooper, S.** ... ... *Surgical Dictionary.* (Ed. by S. A. Lane.) 2 vols. (2) 1861

**Dioscorides, A.** ... *Opera quæ extant omnia.* (Greek and Latin.) (2) 1589
**Dowse, S.** ... ... *A Primer of the Art of Massage* ... ... ... ... 1892

**Edwards, A. Cooper and F. S.** *Diseases of the Rectum and Anus.* 2nd Ed. 1892
**Eminson, T. B. F.** *Epidemic Pneumonia at Scotter* ... ... ... ... 1892
**Ewart, W.** ... ... *Cardiac Outlines* ... ... ... ... ... ... ... ... ... 1892

**Farquharson, A. C.** *Ptomaines and other Animal Alkaloids* ... ... ... 1892
**Freeman, H. W.** ... *The Thermal Baths of Bath* ... ... ... ... (4) 1888

**Galen, C.** ... ... Γαληνοῦ ἅπαντα. 5 vols. in 10 ... ... ... (2) 1538
**Green, T. H.** ... *Pathology and Morbid Anatomy* ... (1) 6th Ed. 1884
**Griffith, A H.** ... *The Diagnosis of Intra-Ocular Growths* ... ... ... 1892

**Harrison, R.** ... *Surgical Anatomy of the Arteries.* 2 vols. 3rd Ed. 1833
*Health: The Voyage to South Africa and Sojourn there* ... ... ... ... 1891
**Hedley, W. S.** ... *Hydro-Electric Methods in Medicine* ... ... ... ... 1892
**Hewson, W.** ... *Works.* (Ed. by G. Gulliver) ... ... ... (1) 1846

**Johnston-Lavis, H. J.** *The Prescribers' Guide to the Harrogate Mineral Waters* ... ... ... ... ... ... ... ... .. ... 1892
**Jones, W. E. Steavenson and H. L.** *Medical Electricity* ... ... ... 1892

**Lœwenberg, Dr...** *L'Otite Grippale...* ... ... ... ... ... ... ... ... ... 1892

**Luff, A. P.** ... ... *A Manual of Chemistry* ... ... ... ... ... ... ... 1892

**Martindale, W....** *The Extra Pharmacopœia* ... ... ... ... ... 7th Ed. 1892
**Moure et J. Bergonié, E. J.** *Du Traitement par l'Électrolyse des Déviations et Éperons de la Cloison du Nez...* ... 1892

**Ridge, J. J.** ... *Alcohol and Public Health* ... ... ... ... ... ... 1892
**Roberts, R.** ... *Observations on Dislocations, &c.* ... ... ... ... ... 1829

**Snell, S.** ... ... *Miners' Nystagmus* ... ... ... ... ... ... ... ... ... 1892
**Steavenson and H. L. Jones, W.E.** *Medical Electricity* ... ... ... 1892
**Syme, J.** ... ... *The Principles of Surgery* ... ... ... ... ... (1) 1832
,, ... ... *On Diseases of the Rectum* ... ... (1) 2nd Ed. 1846

**Todd and W. Bowman, R.B.** *Physiological Anatomy.* (3) 1845; (3) Part III. 1847
**Traill, T. S.** ... *Medical Jurisprudence* ... ... ... (3) 2nd Ed. 1840

**Vogel, J.** ... .. *Pathological Anatomy of the Human Body* ... (3) 1847

---

### TRANSACTIONS, REPORTS, JOURNALS, &c.

*The list of current Periodicals to be seen in the Library is given in the Journal for March.*

American Journal of Obstetrics, The ... (3) Vols. XVII., XVIII. 1884-85
American Journal of the Medical Sciences, The (7) Vol. XCVIII., 1889; Vols. CI.—CIII. 1891-92
American Practitioner and News, The ... ... (7) Vols. IX. and X. 1890
Annals of Surgery ... ... ... ... ... ... ... ... ... (7) Vol. XII. 1890
Annual of the Universal Medical Sciences. 5 vols. ... ... ... .. 1892
Archives de Neurologie ... ... ... ... ... ... Tome XXIII. 1892
Archives of Surgery ... ... ... (7) Vol. I., 1890; Vols. II., III. 1891-92
Asclepiad, The ... ... ... ... ... ... ... ... ... Vol. VIII. 1891
Australasian Medical Gazette, The (7) Vols. VIII., IX., 1889-90; Vol. X. 1891
Australian Medical Journal, The (7) Vol. XII., 1890; Vol. XIII. 1891
Birmingham Medical Review, The (7) Vols. XXVII., XXVIII., 1890; Vols. XXIX.—XXXI. 1891-92
British Gynæcological Journal, The ... ... ... ... Vol. VII. 1891
British Medical Journal ... ... ... ... ... ... ... Vol. I for 1892

Bulletin de l'Academie Royale de Belgique ... ... ... ...Tome V. 1891
Calendar of the Royal College of Surgeons of England ... ... ... 1891
China Medical Missionary Journal, The (6) Vols. II., III. 1888-89
Cincinnati Lancet-Clinic, The ... ... ... (7) Vols. LXIII., LXIV. 1890
Dublin Journal of Medical Science, The (5) Vol. LXXV., 1883;
(7) Vols. LXXXIX., XC., 1890; Vol. XCIII. 1892
Edinburgh Medical Journal ... (7) Part I., Vol. XXXVI., 1891;
Part II., Vol. XXXVII. 1892
Gazette de Gynécologie ... ... ... ... ... ... ... (7) Tome V. 1890
Glasgow Medical Journal, The ... ... ... ... Vol. XXXVII. 1892
Hospitals—
Guy's Hospital Reports. ... ... ... ... Vol. XLVIII. 1892
Hospice de la Salpêtrière. Clinique des Maladies du Système
Nerveux ... ... ... ... ... ... ... ... ... Tome I. 1892
Saint Bartholomew's Hospital Reports—
Index Vols. I.—XX., 1885; Vols. XXIII.—XXVII. 1887-91
St. Thomas's Hospital Reports ... ... ... ... Vol. XX. 1892
Illustrirte Monatsschrift der ärztlichen Polytechnik ... ... ... (7) 1890
Index Medicus ... ... ... ... ... ... ... ... (7) Vol. XII. 1890
Indian Medical Gazette, The ... ... ... ... ... ... Vol. XXVI. 1892
Journal of Nervous and Mental Disease, The ... ... Vol. XVI. 1891
Journal of the American Medical Association, The—
(7) Vols. XIII.—XV., 1889-90; Vols. XVI.—XVIII. 1891-92
Laboratory Reports. Royal College of Physicians, Edinburgh. Vol. IV. 1892
Lancet, The ... ... ... ... (7) Vols. I., II. for 1890; Vol. I. for 1892
London Medical Record, The ... ... ... (3) Vols. XIV., XV. 1886-87
London Medical Recorder, The ... ... ... (3) Vols. I., II. 1888-89
Medical Age, The ... ... (7) Vols. VII., VIII., 1889-90; Vol. IX. 1891
Medical and Surgical Reporter, The (7) Vols. LXII., LXIII., 1890;
Vols. LXIV., LXV. 1891
Medical Chronicle, The ... (7) Vols. XI., XII., 1890; Vol. XV. 1892
Medical News, The ... ... ... ... (7) Vols. LVI., LVII., 1890;
Vols. LVIII., LX. 1891-92
Medical Record ... ... ... (7) Vol. XXXVII., 1890; Vol. XLI. 1892
Medicinische Bibliographie ... ... (7) 1888; (7) 1889; (7) 1890; 1891
Montreal Medical Journal, The ... ... (7) Vol. XVIII., 1890;
Vols. XIX., XX. 1891-92
New York Medical Journal, The (7) Vol. LI., 1890; Vol. LV. 1892
New Zealand Medical Journal, The ... ... ... ... ... Vol. IV. 1891
Retrospect of Medicine, Braithwaite's ... ... ... ... Vol. CV. 1892
St. Louis Medical and Surgical Journal, The Vols. LX.—LXII. 1891-92
Sanitary Record, The ... ... ... ... ... ... ... ... ... (5) 1886
Sei-I-Kwai Medical Journal, The ... ... ...(7) Vols. VIII., IX. 1889-90
Sperimentale, Lo ... ... ... ... .. ... (7) 1889; (7) 1890; 1891
Union Médicale, L' ... ... ... ... ... ... ... ... Tome LIII. 1892

